# CSPα—chaperoning presynaptic proteins

**DOI:** 10.3389/fncel.2014.00116

**Published:** 2014-04-29

**Authors:** Julien Donnelier, Janice E. A. Braun

**Affiliations:** Department of Physiology and Pharmacology, The Hotchkiss Brain Institute, Faculty of Medicine, University of CalgaryCalgary, AB, Canada

**Keywords:** CSP, cysteine string protein, DnaJC5, J protein, chaperones, neurodegeneration, neural differentiation

## Abstract

Synaptic transmission relies on precisely regulated and exceedingly fast protein-protein interactions that involve voltage-gated channels, the exocytosis/endocytosis machinery as well as signaling pathways. Although we have gained an ever more detailed picture of synaptic architecture much remains to be learned about how synapses are maintained. Synaptic chaperones are “folding catalysts” that preserve proteostasis by regulating protein conformation (and therefore protein function) and prevent unwanted protein-protein interactions. Failure to maintain synapses is an early hallmark of several degenerative diseases. Cysteine string protein (CSPα) is a presynaptic vesicle protein and molecular chaperone that has a central role in preventing synaptic loss and neurodegeneration. Over the past few years, a number of different “client proteins” have been implicated as CSPα substrates including voltage-dependent ion channels, signaling proteins and proteins critical to the synaptic vesicle cycle. Here we review the ion channels and synaptic protein complexes under the influence of CSPα and discuss gaps in our current knowledge.

## One J Protein: the total protection plan

Cysteine String Protein (CSPα) is a presynaptic vesicle protein with neuroprotective activity. In humans, mutations in the DNAJC5 gene encoding CSPα that produce heterozygous CSPα point mutations or point deletions cause autosomal-dominant, adult onset neuronal ceroid lipofusinosis (ANCL), a neurodegenerative disorder characterized by accumulation of lysosomal cellular debris (Benitez et al., 2011; Nosková et al., 2011; Velinov et al., 2012). Disease onset is between 20–30 years of age and the course and outcome of ANCL involves, increased anxiety, speech difficulties, ataxia, involuntary movements, seizures, cognitive deterioration, dementia with a shortened life expectancy. In mice, CSPα KO causes activity-dependent synapse loss, progressive defects in neurotransmission and neurodegeneration verifying CSPα’s anti-neurodegenerative function (Fernández-Chacón et al., 2004; García-Junco-Clemente et al., 2010). CSPα heterozygote mice, which have reduced levels of CSPα, are phenotypically normal suggesting that wild type mice normally have “extra” CSPα protection (Fernández-Chacón et al., 2004). In *Drosophila melanogaster*, CSPα KOs that survive to adulthood demonstrate temperature-sensitive paralysis, uncoordinated movement, shaking and early death (Zinsmaier et al., 1994). It is clear that the function of CSPα is to protect the synapse, what is not known is the mechanism(s) underlying the prevention of synapse loss by CSPα. Understandably, much effort has focused on delineating the cellular pathway of CSPα-mediated protection.

## The CSPα trimeric complex

CSPα contains a “J-domain” which is a ∼70-amino acid region of homology shared by bacterial DnaJ and all other J proteins as well as a palmitoylated cysteine-rich “string” region used for membrane attachment to the outer leaflet of synaptic vesicles (Braun and Scheller, 1995). Rather than being “constitutively active” CSPα becomes active upon assembly with SGT (small glutamine-rich tetratricopeptide repeat-containing protein) and Hsc70 (70-kDa heat-shock cognate protein) (Braun et al., 1996; Tobaben et al., 2001). Hsc70 is a central hub of the cellular chaperone network (Craig et al., 2006; Kakkar et al., 2012), and it follows that collapse of Hsc70 would be expected to cause collapse of the J protein network. Each member of the J protein family—there are 49 J proteins in *Homo sapiens—*has a J domain that activates Hsc70’s ATPase activity for conformational work on diverse “client proteins” (Kakkar et al., 2012). Outside of the J domain, J proteins have little, if any, structural similarity. These non-homologous regions are, almost certainly, determinants of J protein specificity but do not provide much clarity into the functionality of specific J proteins. Since other J proteins do not compensate, CSPα is generally viewed as facilitating highly specific folding events. *In vitro*, mutation of the invariant tripeptide of histidine, proline and aspartic acid (HPD motif) located between helices II and III of CSPα’s four α helical J domain creates a loss-of-function mutant that does not activate Hsc70 (Chamberlain and Burgoyne, 1997). CSPα is also found in non-neuronal secretory cells including exocrine (Braun and Scheller, 1995; Zhao et al., 1997; Weng et al., 2009), endocrine (Brown et al., 1998; Zhang et al., 2002) and neuroendocrine (Kohan et al., 1995; Chamberlain et al., 1996) secretory granules and mammary cell small vesicles (Gleave et al., 2001). That said, the reduced life-span in CSPα KO mice is due to neurodegeneration.

## The interval preceding fulminant neurodegeneration

CSPα KO mice appear normal at birth, but around postnatal day 20, they develop progressive motor deficits and CNS degeneration, followed by early lethality between days 40–80 (Fernández-Chacón et al., 2004). In this context it is noteworthy that CSPα is not required for neurotransmitter release but only necessary to maintain synaptic function and architecture. *Which client proteins are critical for triggering the cascade of events leading to degeneration?* The field is now turning to better appreciate the age interval of CSPα KO mice when the demise of the synapse is likely to begin. While this early window remains to be fully dissected, we know that around 20 proteins have altered expression patterns by P28 and that these proteins represent potential primary “misfolding events” (Zhang et al., 2012). Activity-dependent degeneration in mice and temperature-sensitive paralysis in *Drosophila* are distinguishing features of CSPα null models. In mice, synapses that fire frequently, such as those associated with photoreceptors and GABAergic neurons, are lost first (Schmitz et al., 2006; García-Junco-Clemente et al., 2010). Early impairments in motor terminals are characterized by a failure to sustain prolonged release and impaired synaptic vesicle recycling (Rozas et al., 2012). In *Drosophila*, the ∼50% reduction of nerve-evoked neurotransmitter release at 18–22°C and a drastic temperature-sensitive reduction in evoked release above 29°C is well established (Umbach et al., 1994; Zinsmaier et al., 1994; Nie et al., 1999; Dawson-Scully et al., 2000, 2007; Arnold et al., 2004; Bronk et al., 2005). Activity-and temperature-dependent degeneration in CSPα KO reflect the idea that nerve terminals are particularly vulnerable to misfolding and that CSPα is indispensable to refolding and repair. It is likely that CSPα is designed to facilitate folding of multiple “client proteins” whose unfolding leads to a second set of separate downstream degenerative events. However, many questions remain regarding CSPα’s “protein client lineup”.

## “Rescue” of CSPα KO mice

Endogenous J proteins do not serve as “back ups” for the deletion of CSPα, however, overexpression of α-synuclein abrogates motor impairments and lethality in CSPα KO mice while KO of endogenous α-synuclein speeds up neurodegeneration (Chandra et al., 2005). Of note, mice deficient in α-synuclein do not have an obvious phenotype (Chandra et al., 2005). α-synuclein is a soluble presynaptic protein of unknown function that associates with synaptic vesicles and is the major component of Lewy bodies, a landmark of Parkinson’s disease and other neurodegenerative disorders (Maroteaux et al., 1988; Burré et al., 2010). CSPα/α-synuclein do not interact and Hsc70 ATPase is not activated by α-synuclein, hence, while CSPα and α-synuclein may target common “client proteins” they are nonetheless mechanistically distinct (Chandra et al., 2005). Somewhat paradoxically, treatment of CSPα KO mice with proteasome inhibitors ameliorates neurodegeneration and extends the life-span of CSPα KO mice (Sharma et al., 2012b). Thus, the neurodegeneration observed in CSPα KO mice is not due to a reduction in the proteasome capacity and the consequential elevation of ubiquintinated synaptic proteins. While the physiological implications of these findings have not yet been elucidated, rescue of degeneration in CSPα KO mice serves as proof-of-principle for intervention in nerve terminal failure and synapse loss and may pave the way for development of therapeutic agents that prevent neurodegeneration.

## Exocytosis and endocytosis machinery

SNARE (soluble N-ethylmalemide-sensitive factor attachment receptors) proteins are fundamental to presynaptic vesicle-release events and for that reason have been closely scrutinized as possible CSPα “clients”. The t-SNARE, SNAP25 (synaptosomal associated protein of 25 kDa) expression is decreased in CSPα KO mice (Chandra et al., 2005; Zhang et al., 2012). However, it is the effective assembly/disassembly of SNAP25 into the SNARE complex during repeated rounds of exocytosis/endocytosis that correlates with the maintenance of synaptic function rather than cellular levels of SNAP25 (Sharma et al., 2011b, 2012a). While neurodegeneration is rescued by over-expression of wild-type, but not inactive mutants of SNAP-25, the decline in SNAP25 expression *per se* is unlikely to be a primary cause of neurodegeneration, as SNAP25 heterozygous mice with ∼50% reduction in SNAP25 levels are phenotypically normal (Washbourne et al., 2002; Sharma et al., 2011b). Furthermore, the dramatic rescue of neurodegeneration in CSPα KO mice by α-synuclein, rescues the association of SNAP25 with other SNAREs but does not ameliorate the decrease in SNAP-25 expression (Sharma et al., 2011b, 2012a). More recently, it was shown that treatment of CSPα KO mice with proteasome inhibitors reverses impairment of SNARE-complex assembly and alleviates neurodegeneration (Sharma et al., 2012b). CSPα has also been shown to interact with the t-SNARE, syntaxin (Nie et al., 1999; Evans et al., 2001) and the Ca^2+^ binding protein, synaptotagmin (Evans and Morgan, 2002), emphasizing its role in chaperoning the exocytosis machinery. Taken together, degeneration in CSPα KO mice is halted by interventions that correct SNARE complex function including interventions that influence SNARE complex assembly without elevating SNAP25 levels. A separate line of investigation has revealed that CSPα KO mice also have an endocytosis defect resulting in the failure to recycle and maintain the size of the synaptic vesicle pool during prolonged stimulation (Rozas et al., 2012). In fact, early on (P28) in the course of degeneration the GTPase, dynamin 1, which is essential for endocytosis, is reduced by ∼40% in CSPα KO mice (Zhang et al., 2012). CSPα directly interacts with dynamin 1 to promote polymerization, a process required in membrane fission (Zhang et al., 2012), however the mechanistic details linking endocytosis and CSPα dysfunction remain to be established. Insights into exocytosis/endocytosis defects will undoubtedly prove to be important in understanding the pathological uncoupling between presynaptic exocytosis and endocytosis.

## Presynaptic Ion channels: channel proteostasis and multi-Protein Complexes

*Do changes in presynaptic ion channels or ion channel complexes trigger the activity-dependent and temperature-dependent neurodegeneration in CSPα-KO mice?* We have shown that large conductance Ca^2+^-and voltage-activated K^+^ (BK) channels are ∼2.5 fold higher in the brain of CSPα null mice compared with age-matched wild types (Kyle et al., 2013). This increase in expression is observed at an early age (i.e., P23-P27), when levels of neuronal K_v_1.1, K_v_1.2 and Ca_v_2.2 do not change. Physiologically, BK channels are activated by membrane depolarization and/or elevations in intracellular Ca^2+^ and drive the membrane potential towards the K^+^ equilibrium potential. Under normal conditions, BK channels regulate repolarization of the action potential, thereby regulating excitability of neurons, as well as presynaptic neurotransmitter release. Further, ectopic expression of dysfunctional CSPα mutants (i.e., CSPα_HPD-AAA_, CSPα_L116Δ_, CSPα_L115R_) also elicits elevation of BK channel expression and macroscopic current density (Kyle et al., 2013; Ahrendt et al., 2014) suggesting that the observed increase, at least initially, reflects an elevation of functional, rather than mis-folded or aggregated BK channel protein. CSPα_HPD-AAA_ is a loss-of-function mutant in which the essential J domain required for activation of Hsc70ATPase is disrupted but the cysteine string anchor is functional. The increase found in the presence of this loss-of-function mutant is consistent with the increase in BK channel expression observed in CSPα null mice. On the other hand, while CSPα_L116Δ_ and CSPα_L115R_ increase BK channel density at the membrane, the increase is not as large as that observed with CSPα_HPD-AAA_ (Kyle et al., 2013), suggesting that CSPα_L116Δ_ and CSPα_LL115R_ are partial loss-of-function mutants. In humans, deletion of residue 116 or replacement of Lys115 by Arg in the cysteine string region results in ANCL (Benitez et al., 2011; Nosková et al., 2011; Velinov et al., 2012), however the mechanism(s) underlying disease pathology is not known. It is becoming increasingly clear that changes in the cysteine string region can promote oligomerization. Wild type CSPα self-associates (Braun and Scheller, 1995) and this dimerization is eliminated in the absence of the cysteine string region (Xu et al., 2010). When expressed in bacteria, which lack eukaryotic palimitoyltransferase enzymes, CSPα forms oligomers and the cysteine string region is important for the self-association (Swayne et al., 2003). Two mutations in the cysteine string region of CSPα, L116δ and L115R, do not effectively anchor to synaptic vesicles and form oligomers indicating the cysteine string region is closely tied to oligomerization properities (Greaves et al., 2012). Clearly, identification of the neuronal location of CSPα_L116Δ_/CSPα_L115R_ oligomers is central to understanding ANCL disease progression. Thus, disruption of the anchor to the synaptic vesicle while maintaining the functional J domain in the CSPα_L116Δ_ and CSPα_L115R_ mutants and subsequent CSPα oligomerization and cellular mislocalization likely leads to indiscriminate chaperone activity. We speculate that CSPα_L116Δ_ and CSPα_L115R_ cause both a partial loss-of-function (i.e., reduced chaperone activity at the synaptic vesicle) as well as gain-of-function (i.e., increased chaperone activity at a different cellular location). Consistent with this notion, CSPα heterozygote mice do not show neurodegeneration while ANCL patients show adult-onset neurodegeneration most likely due to mislocalized/oligomerized mutant CSPα chaperone activity outside the presynapse. *Do changes in BK channel activity link to neurological disorders or neurodegeneration?* Several studies have reported that alterations in the expression and function of BK channels give rise to neural dysfunction. For example, genetic deletion of BK channel subunits in mice (Sausbier et al., 2004; Brenner et al., 2005) and a gain-of-function channel mutation in humans (Du et al., 2005; Díez-Sampedro et al., 2006) are associated with ataxia and epilepsy, while functional alterations are associated with retardation, schizophrenia and autism (Laumonnier et al., 2006; Zhang et al., 2006; Higgins et al., 2008; Deng et al., 2013). Clearly, when BK channel activity is disrupted, neural excitability and neurotransmitter release are disrupted, but neurodegeneration *per se* does not typically ensue raising a number of interesting questions. *Does the fulminant, activity-dependent neurodegeneration seen in CSPα KO mice result from a coupling of aberrant BK channel expression with synaptic vesicle release/recycling defects? What underlies the rapid rate of degeneration in CSPα KO mice*. *Further, are complexes of BK channels with other synaptic proteins regulated by CSPα?* BK channels are subject to a wide array of regulatory processes, including interactions with SNARE proteins (Ling et al., 2003); however the precise details of these channel complexes in CSPα KO mice remain to be investigated. We speculate that CSPα directly modulates BK channel density, nonetheless this is not a foregone conclusion. Future experiments will undoubtedly establish whether CSPα acts indirectly via one of the known regulators of BK channel activity or by directly targeting the channels.

*Do functional and/or structural changes in voltage-dependent Ca^*2+*^channels occur early in the pathological sequence of events underlying neurodegeneration in CSPα KO mice?* Whether CSPα directly regulates Ca^2+^currents remains contentious. Ca^2+^ entry into presynaptic nerve terminals is fundamental to neurotransmission and consequently subject to multiple levels of control. A large body of work has clarified that neurotransmission defects at the neuromuscular junction involve a decrease in quantal content, a reduction in the calcium sensitivity of evoked exocytosis and anomalous bursts of spontaneous neurotransmitter release (Umbach et al., 1994; Zinsmaier et al., 1994; Dawson-Scully et al., 2000, 2007; Ruiz et al., 2008). Further, recruitment of Ca^2+^ channels (Chen et al., 2002) as well as physical interactions of CSPα with N-type and P/Q type voltage-dependent Ca^2+^ channels have been demonstrated (Leveque et al., 1998; Miller et al., 2003a, b; Swayne et al., 2005, 2006). Whether it is the synaptic vesicle-anchored monomeric CSPα or the mislocalized CSPα oligomers that regulate Ca^2+^currents requires further investigation. We have found that CSPα influences the regulation of N-type Ca^2+^ channels by heterotrimeric GTP binding proteins (Magga et al., 2000; Miller et al., 2003b; Natochin et al., 2005). There are still many unanswered questions regarding the role of CSPα in regulating synaptic channels and synaptic channel complexes. The CSPα KO mouse model offers an excellent opportunity to study channel proteostasis and channel complexes that may contribute to activity-dependent neurodegeneration. How CSPα might function in different secretory cells (e.g., pancreatic exocrine and endocrine cells), given the differences in their complement of ion channels, also remains to be determined. Further efforts will be needed to untangle the neuroprotective pathway(s) involving CSPα at the synapse and to establish precisely the complement of synaptic channels involved in the cascade of neurodegenerative events triggered by the absence of CSPα.

In summary, ion channels are physiologically regulated within tight limits and present evidence implicates the presynaptic molecular chaperone CSPα in the fine-tuning of functional channel levels and regulation of synaptic channel complexes. In the future, it will be interesting to determine whether CSPα is also important for the quality control of mis-folded/aberrant channels. The time course of the expression changes in SNAP25, dynamin 1 and synaptic channels in CSPα KO mice remains a key question.

## Jeopardizing the synapse: links to Alzheimer’s and Parkinson’s disease

Clearly, the dysfunction of CSPα is linked to undesired consequences. Neurodegenerative diseases such as Alzheimer’s disease and Parkinson’s disease show a characteristic loss of neurons and synapses (Muchowski and Wacker, 2005). The identification of CSPα as a central chaperone for the maintenance of synapses raises the questions: *are cellular CSPα-neuroprotective pathways compromised in neurodegenerative disorders other than ANCL? Do functionally impaired proteins that build up in neurodegenerative disorders interfere with CSPα chaperone activity?* It is noteworthy that lysosomal lipofuscin inclusions, like those seen in ANCL, are present in very early onset (age 30) and rapidly progressing Alzheimer’s disease caused by presenilin 1 mutations (Dolzhanskaya et al., 2014). Moreover, CSPα is reduced in the frontal cortex of humans with Alzheimer’s disease (Zhang et al., 2012) and SNARE complex assembly is impaired in human brain tissue from patients with Alzheimer’s Disease and Parkinson’s Disease (Sharma et al., 2012b). Interference with CSPα, SNARE complex assembly, dynamin 1 assembly and ion channel complexes by impaired/toxic proteins typically found in neurodegenerative disorders may, at least in part, contribute to neurodegeneration and these possibilities will undoubtedly be the focus of future investigations.

## Future perspectives

When CSPα is compromised, protein surveillance and proteostasis mechanisms in the neuron fail and the integrity of pre-synaptic terminals is compromised. The field is now poised to tackle in detail the pathogenic sequence of events responsible for activity-dependent neurodegeneration in the absence of CSPα chaperone activity. The emerging detailed molecular blueprint will undoubtedly serve investigators well in the development of therapeutics that protect against synaptic loss in neurodegenerative disorders.

## Conflict of interest statement

The authors declare that the research was conducted in the absence of any commercial or financial relationships that could be construed as a potential conflict of interest.
